# Association of Nonpharmaceutical Interventions During the COVID-19 Pandemic With Invasive Pneumococcal Disease, Pneumococcal Carriage, and Respiratory Viral Infections Among Children in France

**DOI:** 10.1001/jamanetworkopen.2022.18959

**Published:** 2022-06-28

**Authors:** Alexis Rybak, Corinne Levy, François Angoulvant, Anne Auvrignon, Piotr Gembara, Kostas Danis, Sophie Vaux, Daniel Levy-Bruhl, Sylvie van der Werf, Stéphane Béchet, Stéphane Bonacorsi, Zein Assad, Andréa Lazzati, Morgane Michel, Florentia Kaguelidou, Albert Faye, Robert Cohen, Emmanuelle Varon, Naïm Ouldali

**Affiliations:** 1 Association Clinique et Thérapeutique Infantile du Val-de-Marne, Créteil, France; 2 Association Française de Pédiatrie Ambulatoire, Saint-Germain-en-Laye, France; 3 Assistance Publique–Hôpitaux de Paris, Service d'Accueil des Urgences Pédiatriques, Université de Paris, Paris, France; 4 Assistance Publique–Hôpitaux de Paris, Robert Debré University Hospital, Epidémiologie Clinique–Évaluation Économique Appliqué aux Populations Vulnérables, Institut National de la Santé et de la Recherche Médicale, Unité Mixte de Recherche 1123, Université de Paris, Paris, France; 5 Université Paris Est, Institut Mondor de Recherche Biomédicale, Groupe d'Etude de Maladies Infectieuses Néonatales et Infantiles, Créteil, France; 6 Groupe de Pathologie Infectieuse Pédiatrique, Paris, France; 7 Clinical Research Center, Centre Hospitalier Intercommunal de Créteil, Créteil, France; 8 Institut National de la Santé et de la Recherche Médicale, Centre de Recherche des Cordeliers, Unité Mixte de Recherche Scientifique 1138, Université de Paris, Paris, France; 9 Assistance Publique–Hôpitaux de Paris, Service de Pédiatrie Générale, Robert Debré University Hospital, Université de Paris, Paris, France; 10 Direction des Maladies Infectieuses, Santé Publique France, Saint-Maurice, France; 11 Centre National de Référence des Infections Respiratoires, Institut Pasteur, Paris, France; 12 Assistance Publique–Hôpitaux de Paris, Service de Microbiologie, Robert Debré University Hospital, Université de Paris, Paris, France; 13 Infection Antimicrobials Modelling Evolution, Institut National de la Santé et de la Recherche Médicale, Unité Mixte de Recherche 1137, Université de Paris, Paris, France; 14 Service de Pédiatrie Médicale, Centre Hospitalier Universitaire Caen-Normandie, Caen, France; 15 Chirurgie Générale, Digestive et de l'Obésité, Centre Hospitalier Intercommunal de Créteil, Créteil, France; 16 Institut National de la Santé et de la Recherche Médicale, L’Institut Mondor de Recherche Biomédicale, Unité 955, Université Paris-Est Créteil, Créteil, France; 17 Assistance Publique–Hôpitaux de Paris, Centre d’Investigation Clinique 1426, Robert Debré University Hospital, Université de Paris, Paris, France; 18 Néonatalogie et Réanimation Néonatale, Centre Hospitalier Intercommunal de Créteil, Créteil, France; 19 Microbiologie et Centre National de Référence du Pneumocoque, Centre Hospitalier Intercommunal de Créteil, Créteil, France; 20 Service des Maladies Infectieuses Pédiatriques, Centre Hospitalier Universitaire Sainte-Justine, Université de Montréal, Québec, Canada

## Abstract

**Importance:**

An association between pneumococcal nasopharyngeal carriage and invasive pneumococcal disease (IPD) has been previously established. However, it is unclear whether the decrease in IPD incidence observed after implementation of nonpharmaceutical interventions (NPIs) during the COVID-19 pandemic was associated with concomitant changes in pneumococcal carriage and respiratory viral infections.

**Objective:**

To assess changes in IPD incidence after the implementation of NPIs during the COVID-19 pandemic and examine their temporal association with changes in pneumococcal carriage rate and respiratory viral infections (specifically respiratory syncytial virus [RSV] and influenza cases) among children in France.

**Design, Setting, and Participants:**

This cohort study used interrupted time series analysis of data from ambulatory and hospital-based national continuous surveillance systems of pneumococcal carriage, RSV and influenza-related diseases, and IPD between January 1, 2007, and March 31, 2021. Participants included 11 944 children younger than 15 years in France.

**Exposures:**

Implementation of NPIs during the COVID-19 pandemic.

**Main Outcomes and Measures:**

The estimated fraction of IPD change after implementation of NPIs and the association of this change with concomitant changes in pneumococcal carriage rate and RSV and influenza cases among children younger than 15 years. The estimated fraction of change was analyzed using a quasi-Poisson regression model.

**Results:**

During the study period, 5113 children (median [IQR] age, 1.0 [0.6-4.0] years; 2959 boys [57.9%]) had IPD, and 6831 healthy children (median [IQR] age, 1.5 [0.9-3.9] years; 3534 boys [51.7%]) received a swab test. Data on race and ethnicity were not collected. After NPI implementation, IPD incidence decreased by 63% (95% CI, −82% to −43%; *P* < .001) and was similar for non–13-valent pneumococcal conjugate vaccine serotypes with both high disease potential (−63%; 95% CI, −77% to −48%; *P* < .001) and low disease potential (−53%; 95% CI, −70% to −35%; *P* < .001). The overall pneumococcal carriage rate did not significantly change after NPI implementation (−12%; 95% CI, −37% to 12%; *P* = .32), nor did the carriage rate for non-PCV13 serotypes with high disease potential (−26%; 95% CI, −100% to 52%; *P* = .50) or low disease potential (−7%; 95% CI, −34% to 20%; *P* = .61). After NPI implementation, the estimated number of influenza cases decreased by 91% (95% CI, −74% to −97%; *P* < .001), and the estimated number of RSV cases decreased by 74% (95% CI, −55% to −85%; *P* < .001). Overall, the decrease in influenza and RSV cases accounted for 53% (95% CI, −28% to −78%; *P* < .001) and 40% (95% CI, −15% to −65%; *P* = .002) of the decrease in IPD incidence during the NPI period, respectively. The decrease in IPD incidence was not associated with pneumococcal carriage, with carriage accounting for only 4% (95% CI, −7% to 15%; *P* = .49) of the decrease.

**Conclusions and Relevance:**

In this cohort study of data from multiple national continuous surveillance systems, a decrease in pediatric IPD incidence occurred after the implementation of NPIs in France; this decrease was associated with a decrease in viral infection cases rather than pneumococcal carriage rate. The association between pneumococcal carriage and IPD was potentially modified by changes in the number of RSV and influenza cases, suggesting that interventions targeting respiratory viruses, such as immunoprophylaxis or vaccines for RSV and influenza, may be able to prevent a large proportion of pediatric IPD cases.

## Key Points

**Question:**

Was the implementation of nonpharmaceutical interventions (NPIs) during the COVID-19 pandemic associated with changes in the incidence of invasive pneumococcal disease (IPD) and associated pneumococcal carriage and respiratory viral infections (RSVs) in children in France?

**Findings:**

In this cohort study using interrupted time series analysis of data from multiple national surveillance systems involving 11 944 children, the incidence of pediatric IPD decreased after implementation of NPIs during the COVID-19 pandemic. This decrease was associated with decreases in influenza and RSV cases, but the pneumococcal carriage rate remained stable.

**Meaning:**

These results suggest that the established association between pneumococcal carriage and IPD was modified after viral epidemiological changes associated with NPIs, suggesting that interventions targeting respiratory viruses may help prevent a large proportion of pediatric IPD cases.

## Teaser

This cohort study uses data from national continuous surveillance systems to examine the association between nonpharmaceutical interventions for COVID-19 and the incidence of invasive pneumococcal disease, pneumococcal carriage, and respiratory viral infections among children in France.

## Introduction

*Streptococcus pneumoniae* is a major cause of community-acquired invasive bacterial infections worldwide, with more than 300 000 deaths among children aged 1 to 59 months in 2015.^1^ The association between pneumococcal carriage and invasive pneumococcal disease (IPD) has been well described for decades. Nasopharyngeal carriage is a necessary step preceding any pneumococcal disease.^2,3^ All countries monitoring both carriage and IPD in the same population have found an association between carriage and IPD, which is the basis of the disease potential concept, defined as the ability for each serotype to cause disease when that serotype is carried.^4^ Disease potential is consistent from country to country and may be subject to few variations over time.^4,5^ In this context, a review of several simulation studies^6^ proposed surveilling pneumococcal carriage as a proxy to monitor epidemiological change in IPD and assess the outcomes of pneumococcal conjugate vaccine (PCV) implementation.

Viral infections, mainly respiratory syncytial virus (RSV) and influenza, also play a role in triggering pneumococcal disease, including IPD.^7,8^ However, some studies^9,10^ have suggested that this viral coinfection was important for serotypes with low disease potential but less important for serotypes with high disease potential, which may be virulent enough to generate disease without a viral trigger. Thus, the disease potential and impact of PCV implementation with regard to IPD is often estimated without accounting for changes in viral infections. However, the accurate role of viral infections in the ability of pneumococcal serotypes to evolve from colonization to disease has not yet been clarified.

Since March 2020, several mitigation measures have been implemented to reduce the spread of SARS-CoV-2 infection.^11^ A substantial change in viral infections was observed after the implementation of nonpharmaceutical interventions (NPIs), such as social distancing and mask wearing.^12^ In France, the 2020 to 2021 bronchiolitis outbreak was delayed by 3 months, and the peak was reduced by one-half, with no influenza outbreak reported during the year after NPI implementation.^13^ Furthermore, a large decrease in the incidence of IPD was reported worldwide after these measures were implemented.^14^ We therefore aimed to identify, using estimated fraction of change, the changes in IPD incidence after NPI implementation during the COVID-19 pandemic and the association of these changes with concomitant changes in pneumococcal carriage and respiratory viral infections (specifically RSV and influenza cases) among children in France.

## Methods

### Study Design

This cohort study used an interrupted time series analysis of data from several national ambulatory and hospital-based pediatric continuous surveillance systems to analyze IPD incidence, pneumococcal nasopharyngeal carriage in healthy children, serotype distribution among pneumococcal strains isolated from IPD and carriers, and RSV and influenza cases in children. Written informed consent was obtained from parents or legal guardians for the carriage study. This study was approved by the Saint-Germain-en-Laye Ethics Committee. Because other surveillance systems (eg, the National Reference Centre for Pneumococci [NRCP] and RSV and influenza surveillance systems) are part of ongoing surveillance programs coordinated by Santé Publique France, the national public health agency, ethics committee approval and written or verbal informed consent were not required according to the French regulatory authorities (the Commission Nationale de l'Informatique et des Libertés [CNIL] and the Comité d'Expertise pour les Recherches, les Études et les Évaluations dans le Domaine de la Santé [CESREES]). This study followed the Strengthening the Reporting of Observational Studies in Epidemiology (STROBE) reporting guideline.

### Study Data and Settings

#### Invasive Pneumococcal Disease Incidence

The data for IPD incidence from January 1, 2007, to March 31, 2021, were extracted from the French hospital medical information database (Programme de Medicalisation des Systèmes d’Information [PMSI]), an exhaustive national medicoadministrative database that includes all inpatients admitted to any public or private hospital in France.^15^ Diagnoses were coded according to the *International Classification of Diseases, Tenth Revision* (*ICD-10*). An IPD case in a child younger than 15 years was defined as a hospitalization with a primary or secondary diagnosis of pneumococcal meningitis (*ICD-10* code G001) or pneumococcal bacteremia (*ICD-10* code A403). We collected data on age, sex, dates of hospitalization, and deaths. We used the age-specific French population obtained from the National Institute for Statistics and Economic Studies as the denominator to calculate the IPD incidence per 100 000 children.^16^

#### Pneumococcal Carriage in Healthy Children

Between November 1, 2006, and April 30, 2021, we collected data from national continuous surveillance systems involving 77 pediatricians from all French metropolitan regions who obtained nasopharyngeal swabs from healthy children 6 months and older.^17^ Children were excluded if they received antibiotic treatment within 7 days before the swab test. Deep nasopharyngeal samples were obtained and analyzed at the NRCP (Créteil, France) and Robert Debré Hospital (Paris, France). Details on microbiological investigations were published previously.^18,19^ Data collected included medical history and receipt of antibiotic treatment during the 3 months before inclusion.

#### Influenza and RSV Surveillance Systems

We used influenza-like illness as a proxy for influenza cases.^20^ Data were obtained between January 1, 2015, and March 31, 2021, from the Organisation de la Surveillance Coordonnées des Urgences (OSCOUR) network, a national continuous surveillance study.^21^ The OSCOUR gathers data from more than 90% of emergency department visits in France. The number of influenza-like illnesses in children younger than 15 years is transmitted each week to Santé Publique France.

Data on RSV were obtained during the same period from the Réseau National de Laboratoires Hospitaliers surveillance network,^22^ a national continuous surveillance system. Approximately 40 hospital-based laboratories in France reported the weekly number of tests performed and the test results for RSV detection during the RSV epidemic period. All nasopharyngeal tests from both outpatient and hospitalized patients are included without age restriction. These data are transmitted to Santé Publique France and used for weekly epidemiological reports. We also extracted the number of pediatric emergency department visits for bronchiolitis from the OSCOUR network to conduct a sensitivity analysis based on RSV-related disease.

### Microbiological Serotyping

Each year, IPD strains isolated in children younger than 15 years are sent to the NRCP for antimicrobial testing and serotyping.^23^ All *S pneumoniae* strains isolated from healthy carriers and individuals with IPD were serotyped by the NRCP using latex agglutination with antiserum samples provided by the Statens Serum Institute (Copenhagen, Denmark). Serotypes 15B and 15C were considered a single serotype (15B/C) because their capsule is quickly interchangeable.^24^ Based on data in the literature, serotypes 8, 10A, 12F, 22F, 24F, 33F, and 38 were considered non–13-valent PCV (PCV13) serotypes with high disease potential.^5^ Serotypes 6C, 11A, 15A, 15B/C, 17F, 19F, 21, 23A, 23B, 31, 34, 35B, 35F, and nontypeable were considered non-PCV13 serotypes with low disease potential.^5^

### National Nonpharmaceutical Interventions

Since 2003, the 7-valent PCV (PCV7) has been recommended in France. In 2010, the PCV7 was replaced by the PCV13 for all children younger than 2 years without a catch-up vaccination schedule. Vaccination coverage with PCV13 remained greater than 90% during the study period.^25^ Between March 17 and May 11, 2020, a strict lockdown was implemented to reduce the spread of COVID-19.^11^ The lockdown was gradually followed by various mitigation strategies, including social distancing, wearing of face masks by adults and children older than 6 years, and a night curfew. Details of the NPIs implemented in France were provided by the European Centre for Disease Prevention and Control (eTable 1 and eFigure 1 in the Supplement).^11^

We organized the study period into 4 intervals: (1) the PCV7 period from November 1, 2006, to May 31, 2010; (2) the early PCV13 period from June 1, 2011, to December 31, 2014; (3) the late PCV13 period from January 1, 2015, to February 29, 2020; and (4) the NPI period from April 1, 2020, to April 30, 2021. We divided the PCV13 period into early and late categories based on a previously published study^12^ that reported a recurrence of increased IPD incidence in children because of serotype replacement since January 2015 in France.

### Outcome Measures

The primary outcome was the estimated fraction of change in IPD incidence after implementation of NPIs and the association of this change with concomitant changes in pneumococcal carriage rate and the number of respiratory viral infections (specifically influenza and RSV cases) among children younger than 15 years. The secondary outcomes were (1) the monthly incidence of IPD over time among children younger than 15 years, (2) the monthly incidence of IPD associated with serotypes with high and low disease potential over time among children younger than 15 years, (3) the monthly pneumococcal carriage rate over time among healthy children younger than 15 years, (4) the monthly pneumococcal carriage rate of serotypes with high and low disease potential over time among healthy children younger than 15 years, (5) the estimated fraction of IPD change after implementation of NPIs among children who had serotypes with high and low disease potential that was associated with pneumococcal carriage rate and respiratory viral infections (ie, RSV and influenza cases) among children younger than 15 years, (6) the monthly incidence of influenza over time among children younger than 15 years, and (7) the monthly incidence of bronchiolitis over time among children younger than 2 years. For the primary outcome, we used data from January 1, 2007, to March 31, 2021. For the secondary outcomes, we decided to present all of the data available for IPD incidence (from January 1, 2007, to March 31, 2021), the numbers of RSV and influenza cases (from January 1, 2015, to 31 March, 2021), and the pneumococcal carriage rate (from November 1, 2006, to April 30, 2021).

We also conducted a specific analysis for serotype 24F because it has been the leading nonvaccine serotype involved in IPD in France since 2015.^23^ As a control outcome, we analyzed the monthly incidence of *Escherichia coli* invasive disease over time among children younger than 15 years because NPI implementation was not expected to have consequences for this disease.

### Statistical Analysis

First, we built a segmented linear regression model with autoregressive error to estimate the change in IPD incidence after NPI implementation.^26,27^ This model accounted for autocorrelation, seasonality, and temporal patterns before and after the NPIs were implemented. We accounted for seasonality by using an additive model.^28^ We used an autoregressive moving-average term to account for the remaining autocorrelation. According to the literature,^5^ we hypothesized that NPI implementation would have immediate consequences for IPD incidence. Therefore, we included a dummy variable in the model estimating the immediate change after the intervention.^28^ The time unit was set at 1 month. March 2020 was considered a transitional period because NPI implementation started in the middle of this month.

Second, we used the same segmented linear regression model with autoregressive error to estimate the change in pneumococcal carriage rate and the number of RSV and influenza cases after NPI implementation. Third, to estimate the change in IPD associated with concomitant changes in pneumococcal carriage rate and the number of RSV and influenza cases, we fitted a quasi-Poisson regression model including seasonality by using harmonic terms (sines and cosines with 6-month and 12-month periods) to account for seasonality and RSV cases, influenza cases, and pneumococcal carriage rate as explanatory variables.^7^ We then estimated the change in IPD if RSV cases, influenza cases, or pneumococcal carriage rate had remained unchanged during the NPI period by using the same equation and set for influenza (eFigure 2 in the Supplement), RSV (eFigure 3 in the Supplement), and pneumococcal carriage that was used to measure patterns in the pre-NPI period. For each postintervention time point, we compared the forecasted IPD incidence, including the incidence of RSV, influenza, or pneumococcal carriage that remained unchanged, with the fitted IPD incidence. The estimated fraction of IPD change associated with pneumococcal carriage, RSV, or influenza was calculated as the incidence of IPD associated with carriage, RSV, or influenza divided by the fitted incidence of IPD during the NPI period.

 In addition, we performed several sensitivity analyses. The first analysis used a quasi-Poisson regression model that included harmonic terms with only 12-month periods, and the second analysis used a quasi-Poisson regression model that included the number of pediatric emergency department visits for bronchiolitis rather than the percentage of RSV-positive tests over time.

All statistical tests were 2-sided, with *P* = .05 considered statistically significant. The validity of the segmented regression model was assessed by visual inspection of correlograms and analysis of residuals (eFigure 4 and eFigure 5 in the Supplement). All statistical analyses were performed using R software, version 4.0.3 (R Foundation for Statistical Computing), and Stata software, version 15.1 (StataCorp Ltd).

## Results

### Invasive Pneumococcal Disease Incidence

Between January 1, 2007, and March 31, 2021, 5113 children (median [IQR] age, 1.0 [0.6-4.0] years; 2959 boys [57.9%] and 2154 girls [42.1%]) with IPD were identified across all periods (including the transition periods). Data on race and ethnicity were not collected. There were 1445 IPD cases during the PCV7 period, 1031 during the early PCV13 period, 1987 during the late PCV13 period, and 194 during the NPI period. Additional characteristics are shown in Table 1.

**Table 1.** Characteristics of Children With Invasive Pneumococcal Disease in the French Hospital Medical Information Database

Characteristic	Patients, No. (%)						
	PCV7 period^a^	Transition period 1^b^	PCV13 period		Transition period 2^e^	NPI period^f^	All study periods, including transition periods^g^
			Early^c^	Late^d^			
Total patients, No.	1445	416	1031	1987	40	194	5113
Age, median (IQR), y^h^	1.0 (0.6-4.0)	1.0 (0.7-4.0)	2.0 (0.6-5.0)	1.0 (0.6-4.0)	1.0 (0.5-2.0)	1.0 (0.6-3.0)	1.0 (0.6-4.0)
Sex							
Male	853 (59.0)	231 (55.5)	575 (55.8)	1170 (58.9)	22 (55.0)	108 (55.7)	2959 (57.9)
Female	592 (41.0)	185 (44.5)	456 (44.2)	817 (41.1)	18 (45.0)	86 (44.3)	2154 (42.1)
Meningitis	703 (48.7)	211 (50.7)	560 (54.3)	1029 (51.8)	23 (42.5)	72 (37.1)	2598 (50.8)
Bacteremia without meningitis	742 (51.3)	205 (48.3)	471 (45.7)	958 (48.2)	17 (57.5)	122 (62.9)	2515 (49.2)
Deaths	60 (4.2)	30 (7.2)	56 (5.4)	68 (3.4)	1 (2.5)	6 (3.1)	221 (4.3)

Abbreviations: NPI, nonpharmaceutical intervention; PCV7, 7-valent pneumococcal conjugate vaccine; PCV13, 13-valent pneumococcal conjugate vaccine.

^a^ The PCV7 period was from January 1, 2007, to May 31, 2010.

^b^ Transition period 1 was from June 1, 2010, to May 31, 2011.

^c^ The early PCV13 period was from June 1, 2011, to May 31, 2014.

^d^ The late PCV13 period was from June 1, 2014, to February 29, 2020.

^e^ Transition period 2 was from March 1 to 31, 2020.

^f^ The NPI period was from April 1, 2020, to March 31, 2021.

^g^ Includes all periods from June 1, 2010, to May 31, 2021 (including transition periods).

^h^ Age was only available in rounded-down years for children older than 1 year in the French hospital medical information database (Programme de Medicalisation des Systèmes d’Information).

**Figure 1.**
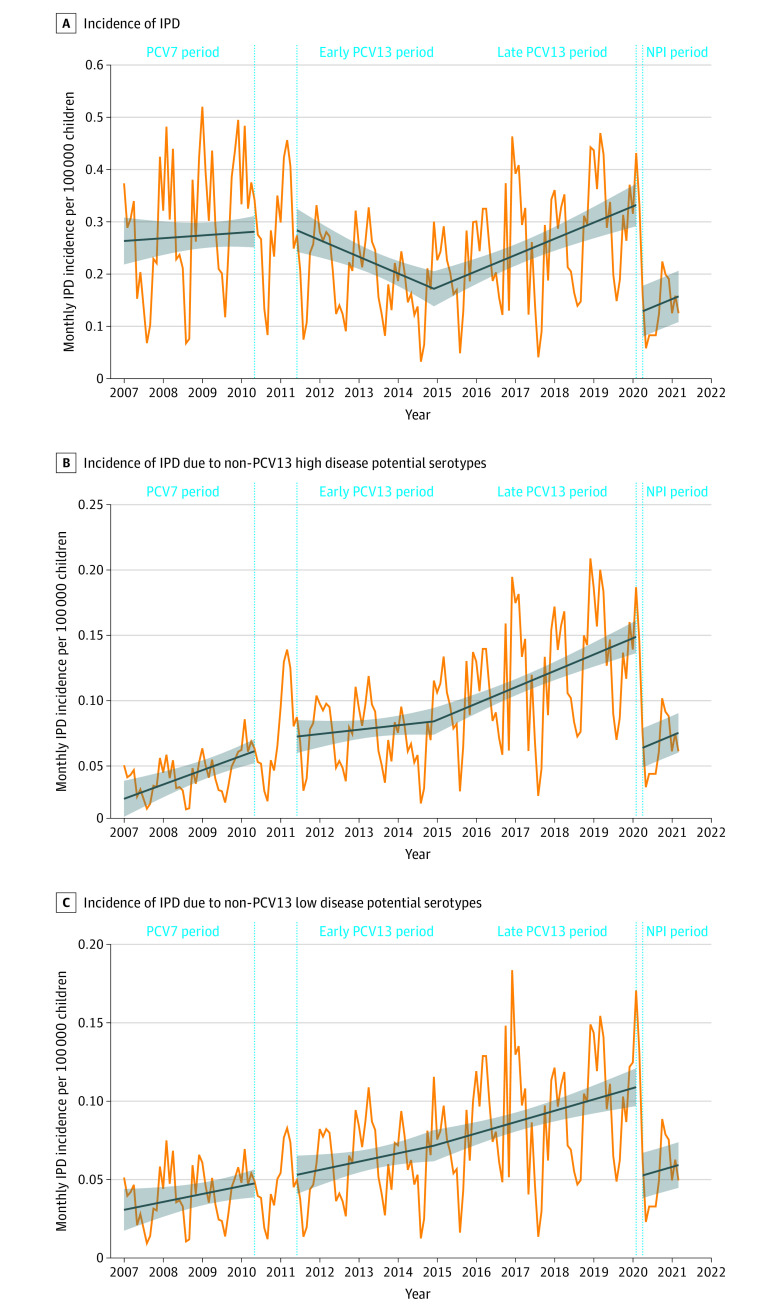
Association of Nonpharmaceutical Intervention (NPI) Implementation With Invasive Pneumococcal Disease (IPD) Incidence per 100 000 Children Younger Than 15 Years The 7-valent pneumococcal conjugate vaccine (PCV7) period was from January 1, 2007, to May 31, 2010; the early 13-valent PCV (PCV13) period, June 1, 2011, to May 31, 2014; the late PCV13 period, June 1, 2014, to February 29, 2020; and the NPI period, April 1, 2020, to March 31, 2021. The blue slope lines were estimated using a segmented regression model. The shaded areas show the 95% CIs estimated using the segmented regression model. The vertical dotted lines show the transition period from PCV13 implementation (April 1, 2010, to May 31, 2011) to NPI implementation (March 1-31, 2020). A, A total of 5113 children were included in the analysis. B, IPD associated with non-PCV13 serotypes with high disease potential (including serotypes 8, 10A, 12F, 22F, 24F, 33F, and 38). A total of 1594 children were included in the analysis. C, IPD associated with non-PCV13 serotypes with low disease potential (including serotypes 6C, 11A, 15A, 15B/C, 17F, 19F, 21, 23A, 23B, 31, 34, 35B, 35F, and nontypeable). A total of 1363 children were included in the analysis.

After NPI implementation, IPD incidence immediately decreased (−63%; 95% CI, −82% to −43%; *P* < .001) (Figure 1A). This IPD decrease was similar among children who had non-PCV13 serotypes with high disease potential (−63%; 95% CI, −77% to −48%; *P* < .001) and low disease potential (−53%; 95% CI, −70% to −35%; *P* < .001) (Figure 1B and C). We found a similar decrease in IPD among children with the 24F serotype (−68%; 95% CI, −84% to −52%; *P* < .001) (eFigure 6 in the Supplement). During the study period, serotyping was performed for 4412 cases in the NRCP. During the NPI period, the most prevalent serotypes involved in IPD were 24F (14 children [17.3%]), 10A (11 children [13.6%]), 23B (9 children [11.1%]), 15B/C (7 children [8.6%]), and 11A (5 children [6.2%]). In comparison, during the late PCV13 period, the most prevalent serotypes were 24F (207 children [15.6%]), 15B/C (102 children [7.7%]), 10A (89 children [6.7%]), 12F (86 children [6.5%]), and 8 (74 children [5.6%]) (eTable 2 in the Supplement).

### Influenza and RSV Cases

After NPI implementation, we estimated that the number of influenza cases decreased by 91% (95% CI, −74% to −97%; *P* < .001). The number of RSV cases decreased by 74% (95% CI, −55% to −85%; *P* < .001).

### *E coli* Invasive Disease Incidence

Between January 1, 2007, and March 31, 2021, 13 234 children (median [IQR] age, 1.0 [0.2-5.0] years; 7374 boys [55.7%] and 5860 girls [44.3%]) with *E coli* invasive disease were identified in the PMSI database. We observed no significant change in *E coli* invasive disease after NPI implementation (−8%; 95% CI, −21% to 5%; *P* = .21) (eFigure 7 in the Supplement).

### Pneumococcal Carriage

**Figure 2.**
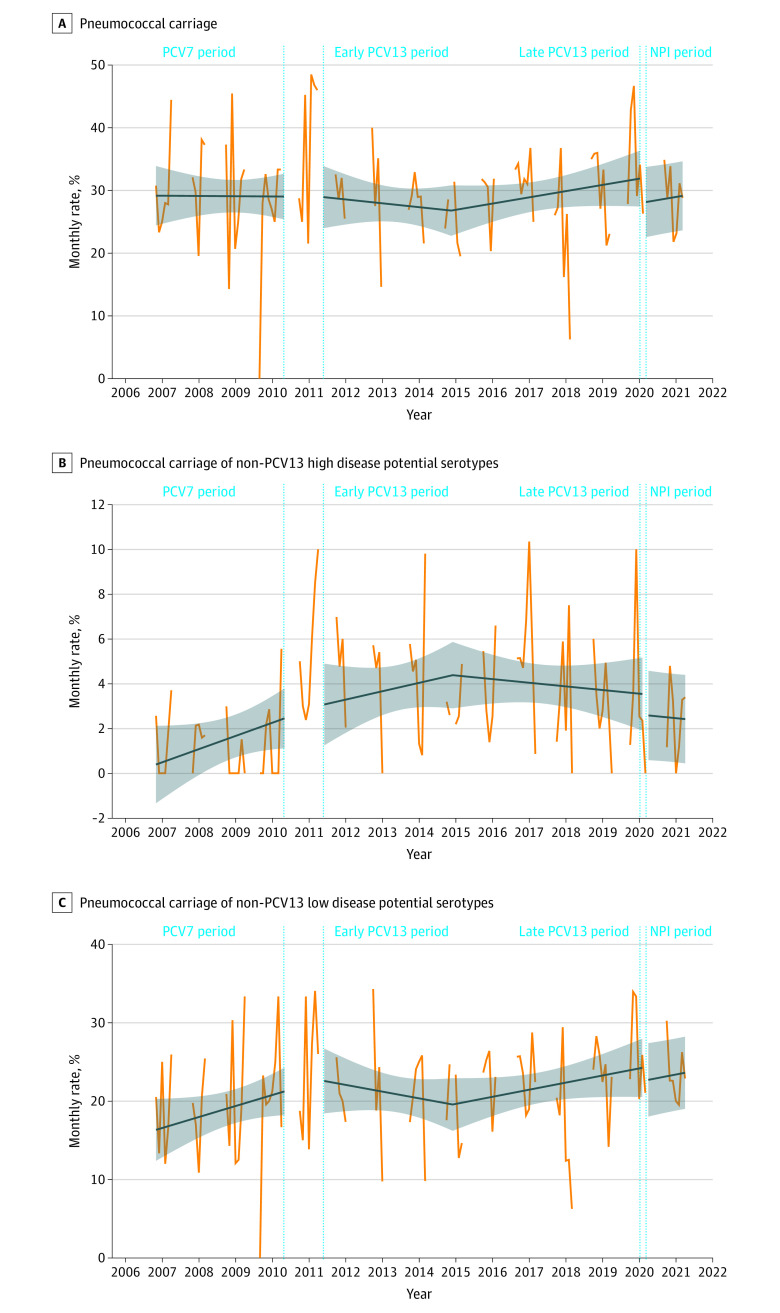
Association of Nonpharmaceutical Intervention (NPI) Implementation With Pneumococcal Carriage in Healthy Children A total of 6831 children were healthy during the study period. The 7-valent pneumococcal conjugate vaccine (PCV7) period was from January 1, 2007, to May 31, 2010; the early 13-valent PCV (PCV13) period, June 1, 2011, to May 31, 2014; the late PCV13 period, June 1, 2014, to February 29, 2020; and the NPI period, April 1, 2020, to March 31, 2021. The blue slope lines were estimated using a segmented regression model. The shaded areas show the 95% CIs estimated using the segmented regression model. The vertical dotted lines show the transition period from PCV13 implementation (April 1, 2010, to May 31, 2011) to NPI implementation (March 1-31, 2020). A, A total of 2013 children were included in the analysis. B, Pneumococcal carriage rates of non-PCV13 serotypes with high disease potential (including serotypes 8, 10A, 12F, 22F, 24F, 33F, and 38). A total of 225 children were included in the analysis. C, Pneumococcal carriage rates of non-PCV13 serotypes with low disease potential (including serotypes 6C, 11A, 15A, 15B/C, 17F, 19F, 21, 23A, 23B, 31, 34, 35B, 35F, and nontypeable). A total of 1455 children were included in the analysis.

Between November 1, 2006, and April 30, 2021, 6831 healthy children (median [IQR] age, 1.5 [0.9-3.9] years; 3534 boys [51.7%] and 3297 girls [48.3%]) received a swab test. Of those, 2013 children (29.5%) across all periods (including the transition periods) carried *S pneumoniae*. The carriage rates were 356 of 1204 children (29.6%) during the PCV7 period, 412 of 1462 children (28.2%) during the early PCV13 period, 920 of 3118 children (29.5%) during the late PCV13 period, and 177 of 610 children (29.0%) during the NPI period. Additional characteristics are shown in Table 2. The pneumococcal carriage rate did not significantly change after NPI implementation (−12%; 95% CI, −37% to 12%; *P* = .32) (Figure 2A), nor did the carriage rates for non-PCV13 serotypes with high disease potential (−26%; 95% CI, −100% to 52%; *P* = .50) or low disease potential (−7%; 95% CI, −34% to 20%; *P* = .61) (Figure 2B and C), especially serotype 24F (−29%; 95% CI, −100% to 66%; *P* = .54) (eFigure 8 in the Supplement).

**Table 2.** Characteristics of Healthy Children Included in the Pneumococcal Carriage Study^a^

Characteristic	Patients, No. (%)						
	PCV7 period^b^	Transition period 1^c^	PCV13 period		Transition period 2^f^	NPI period^g^	All study periods, including transition periods^h^
			Early^d^	Late^e^			
Total patients, No.	1204	417	1462	3118	20	610	6831
Age, median (IQR), y	1.0 (0.7-1.4)	1.3 (0.8-2.0)	1.4 (0.9-2.9)	2 (1.0-6.0)	2.9 (1.2-7.6)	1.9 (0.9-6.1)	1.5 (0.9-3.9)
Sex							
Male	591 (49.1)	215 (51.5)	778 (53.2)	1607 (51.5)	10 (50.0)	333 (54.6)	3534 (51.7)
Female	613 (50.9)	202 (48.5)	684 (46.8)	1511 (48.5)	10 (50.0)	277 (45.4)	3297 (48.3)
Antibiotic prescription in the last 3 mo before inclusion	252 (20.9)	66 (15.8)	201 (13.7)	460 (14.8)	5 (25.0)	52 (8.5)	1036 (15.2)
Nasopharyngeal pneumococcal carriage	356 (29.6)	142 (34.0)	412 (28.2)	920 (29.5)	6 (30.0)	177 (29.0)	2013 (29.5)

Abbreviations: NPI, nonpharmaceutical intervention; PCV7, 7-valent pneumococcal conjugate vaccine; PCV13, 13-valent pneumococcal conjugate vaccine.

^a^ Includes data from analysis of entire database. For pneumococcal carriage, data were available until May 2021. For IPD, data were available until April 2021. For estimated fraction of change, both databases were used simultaneously, so only data until May 2021 were included.

^b^ The PCV7 period was from November 1, 2006, to May 31, 2010.

^c^ Transition period 1 was from June 1, 2010, to May 31, 2011.

^d^ The early PCV13 period was from June 1, 2011, to May 31, 2014.

^e^ The late PCV13 period was from June 1, 2014, to February 29, 2020.

^f^ Transition period 2 was from March 1 to 31, 2020.

^g^ The NPI period was from April 1, 2020, to April 30, 2021.

^h^ Includes all periods from June 1, 2010, to April 30, 2021 (including transition periods).

### Estimated Fraction of Change in Invasive Pneumococcal Disease

We estimated that 53% (95% CI, 28%-78%; *P* < .001) of the decrease in IPD during the NPI period was associated with the decrease in the number of influenza cases, and 40% (95% CI, 15%-65%; *P* < .002) of the decrease was associated with the decrease in the number of RSV cases. The decrease in IPD was not associated with the pneumococcal carriage rate, with carriage accounting for only 4% (95% CI, −7% to 15%; *P* = .49) of the decrease. We found similar results for IPD among children who had non-PCV13 serotypes with high disease potential (influenza: 53% [95% CI, 28%-78%; *P* < .001]; RSV: 41% [95% CI, 15%-66%; *P* = .002]; pneumococcal carriage: 0.4% [95% CI, −4% to 5%; *P* = .86]), non-PCV13 serotypes with low disease potential (influenza: 56% [95% CI, 30%-82%; *P* < .001]; RSV: 38% [95% CI, 12%-63%; *P* = .004]; pneumococcal carriage: 3% [95% CI, −5% to 11%; *P* = .44]), and serotype 24F (influenza: 54% [95% CI, 26%-81%; *P* < .001]; RSV: 37% [95% CI, 9%-65%; *P* = .01]; pneumococcal carriage: 2% [95% CI, −15% to 20%; *P* = .77]) (Table 3). Similar results were also found in the sensitivity analyses (eTable 3 in the Supplement).

**Table 3.** Estimated Fraction of Change in IPD Associated With Changes in Influenza, RSV, and Pneumococcal Carriage After Implementation of Nonpharmaceutical Interventions

Serotype	Estimated fraction of IPD					
	Influenza		RSV		Pneumococcal carriage	
	% (95% CI)	*P* value	% (95% CI)	*P* value	% (95% CI)	*P* value
Overall IPD	53 (28 to 78)	<.001	40 (15 to 65)	.002	4 (−7 to 15)	.49
IPD associated with non-PCV13 serotype						
High disease potential	53 (28 to 78)	<.001	41 (15 to 66)	.002	0.4 (−4 to 5)	.86
Low disease potential	56 (30 to 82)	<.001	38 (12 to 63)	.004	3 (−5 to 11)	.44
IPD associated with 24F serotype	54 (26 to 81)	<.001	37 (9 to 65)	.01	2 (−15 to 20)	.77

Abbreviations: IPD, invasive pneumococcal disease; PCV13, 13-valent pneumococcal conjugate vaccine; RSV, respiratory syncytial virus.

## Discussion

This cohort study assessed the association between pneumococcal carriage and IPD since the implementation of NPIs designed to reduce the spread of COVID-19. We observed a decrease in IPD incidence, with no significant concomitant change in overall pneumococcal carriage or in carriage of non-PCV13 serotypes with low and high disease potential, including serotype 24F. The main factor associated with the decrease in IPD incidence was the decrease in the number of influenza and RSV cases.

Capsular polysaccharides, which define pneumococcus serotypes, are the main factors associated with the ability to cause disease.^29^ The disease potential of a serotype was thought to be subject to limited variation over time, which led a simulation study^30^ to predict a reduction in pneumococcal carriage and IPD incidence after NPI implementation. Using interrupted time series models, we estimated that approximately 90% of the decrease in IPD incidence was associated with decreases in the number of RSV and influenza cases, with no role for pneumococcal carriage. Taken together, these findings suggest that the disease potential of pneumococcal serotypes may be associated with changes in RSV and influenza incidence, both for serotypes with historically high and low disease potential.

For the past 20 years, Israel and France have shared a similar surveillance network for pneumococcal carriage,^18,19,31^ which has allowed for the study of pneumococcal carriage and IPD over time. A recent study from Israel^32^ suggested that pneumococcal carriage rates remained stable during NPI implementation, whereas IPD case numbers substantially decreased during the same period, which was consistent with our results. The IPD reduction was temporally associated with decreases in RSV, influenza, and human metapneumovirus cases, whereas rates of rhinovirus, adenovirus, and parainfluenza cases remained stable.^32^ Thus, epidemiological studies^4,5^ reporting an association between pneumococcal carriage and IPD have assumed that respiratory viral infection cases remain stable, which may not be true in the context of human interventions. Data from the present study suggest that assessing the impact of further public health interventions, such as next-generation PCV implementation, requires both surveillance of pneumococcal carriage and IPD as well as viral epidemiological factors to fully understand the mechanisms underlying changes in IPD incidence.

In our study, the pneumococcal nasopharyngeal carriage rate remained stable despite unprecedented mitigation measures.^11^ Several points should be considered. First, NPIs were mainly focused on adults and children older than 6 years, in whom pneumococcal carriage is less frequent.^33^ Unlike in many other countries, the total duration of daycare center and kindergarten closures in France was short during the NPI period.^34^ This brief closure may have allowed for pneumococcal transmission between young children. However, the incidence of other childhood viral infections, such as varicella, was also substantially reduced in France over the same period, which suggests that NPI implementation also altered contact and transmission between young children.^35^ Second, this stable pneumococcal carriage rate may be a consequence of increased carriage duration combined with decreased transmission. An important competition exists between pneumococcal serotypes, and colonization by a serotype may lead to clearance of a preexisting colonizer.^36^ Thus, in a context of reduced contact between children, serotypes may be less challenged by other serotypes, thereby increasing the carriage duration. If specific studies are required to explore this hypothesis, our results highlight that carriage of *S pneumoniae* is unavoidable in young children, with the virus maintaining an ecological niche that remains occupied despite unprecedented human interventions. This finding has implications for future interventions, such as PCV implementation, that aim to modify pneumococcal carriage and provides new insight into the complex phenomenon of serotype replacement in carriage.

The substantial decrease in viral infections during NPI implementation has led to the immune debt hypothesis, which suggests that reduced natural immunity of the population to respiratory pathogens after NPIs may predispose to larger outbreaks in the future.^13,35^ This suggestion has been supported by findings from a simulation study^37^ and may be confirmed by the recent epidemiological patterns observed in several countries.^13^ Our findings regarding the unchanged ecological niche of *S pneumoniae* suggest no decrease in natural immunity to pneumococcal serotypes. However, given the substantial consequences of RSV and influenza for IPD changes that we highlighted, a larger influenza or RSV outbreak may produce an increase in IPD incidence after relaxation of NPIs, despite unchanged pneumococcal carriage rates.

### Limitations

This study has several limitations. First, our model relies on temporal association, and we cannot establish a causative relationship. Some previous studies using databases^7,8,38^ have supported the role of preceding respiratory viral infection in the occurrence of IPD. Other factors, such as host,^39^ meteorological,^40^ or human activity factors, which have also changed after NPI implementation, were not included in this study. Second, we only assessed the fraction of IPD change associated with concomitant changes in RSV and influenza cases because they have historically been suspected to trigger pneumococcal infections.^7,8^ Other respiratory viruses, such as human metapneumovirus and parainfluenza, may also play a role. It has been reported that the rhinovirus positivity rate remained stable in France after NPI implementation.^41^ Other respiratory viruses are not monitored in France,^42^ and their epidemiological activity could not be included in our model.

Third, the children included in our carriage study were young (median [IQR] age, 1.5 [0.9-3.9] years) and may have been less likely than older children to experience consequences from the NPIs implemented in France. We cannot exclude the possibility that the pneumococcal carriage rate changed in older children. However, associations between pneumococcal carriage in children younger than 5 years, children aged 5 to 17 years, and adults^33^ may play a marginal role in this ecological niche because the carriage rate is less than 10% in this population. Furthermore, pneumococcal serotype changes over time were not assessed individually, and we cannot exclude the possibility that a decrease in acquisition of new pneumococcal serotypes may be involved in the decrease in IPD incidence. Fourth, in our main model, RSV cases were based on the proportion of RSV-positive tests, and testing policies may have changed during the COVID-19 pandemic. However, our sensitivity analysis using visits to bronchiolitis-related pediatric emergency departments as a proxy for RSV cases yielded similar results. Other European countries reported similar findings, with a near absence of influenza cases^43^ and a delayed and reduced RSV epidemic.^44^

Fifth, the PMSI database does not include data on pneumococcal serotypes. Nevertheless, most of the strains isolated from IPD in children were sent for analysis to the NRCP even after NPI implementation. Sixth, the carriage study used only standard culture methods to identify pneumococci, but PCR tests may be more sensitive.^45^ However, we used the same microbiological methods over the study period. Seventh, we cannot exclude the possibility that a fraction of the decrease in IPD incidence may be associated with an avoidance of the pediatric emergency department or a change in criteria for hospital admission. We observed no change in *E coli* invasive disease incidence (used as a control outcome) after NPI implementation, and a similar decrease in IPD incidence^14^ has been reported globally.

## Conclusions

In this cohort study, a decrease in IPD incidence was found among children in France after NPI implementation during the COVID-19 pandemic. This decrease in IPD was associated with decreases in the number of RSV and influenza cases; however, the pneumococcal carriage rate remained largely unchanged. Several RSV vaccines and methods of immunoprophylaxis for RSV infections are currently being developed, and influenza vaccines are available. The findings of this study suggest that targeting these viral pathogens may be able to prevent a large proportion of IPD cases without creating gaps in the pneumococcal ecological niche, which is the main limitation of current PCVs.

## Notes

Supplement. **eTable 1.** Main Nonpharmaceutical Interventions Implemented in France Against the Spread of SARS-CoV-2 During the Study Period **eTable 2.** Serotype Distribution Among *Streptococcus pneumoniae* Strains Isolated from IPD Cases and Carriage in Children **eTable 3.** Sensitivity Analyses of the Estimated Fraction of IPD Change After Nonpharmaceutical Interventions Associated With Changes in RSV, Influenza, and Pneumococcal Carriage **eFigure 1.** Association of Nonpharmaceutical Intervention Implementation and Incidence of Pediatric Invasive Pneumococcal Disease in Children Younger Than 15 Years **eFigure 2.** Association of Nonpharmaceutical Intervention Implementation and Incidence of Pediatric Emergency Visits for Influenza-like Illness in Children Younger Than 15 Years **eFigure 3.** Association of Nonpharmaceutical Intervention Implementation and Percentage of RSV-Positive Tests **eFigure 4.** Autocorrelation Function of Residuals, Partial Autocorrelation Function of Residuals, Standardized vs Fitted Values, and Normal Q-Q Plot of the Model Used to Estimate Change in IPD Incidence After Nonpharmaceutical Intervention Implementation **eFigure 5.** Autocorrelation Function of Residuals, Partial Autocorrelation Function of Residuals, Standardized vs Fitted Values, and Normal Q-Q Plot of the Model Used to Estimate Change in Pneumococcal Carriage After Nonpharmaceutical Intervention Implementation **eFigure 6.** Association of Nonpharmaceutical Intervention Implementation With IPD Incidence Associated With Serotype 24F per 100 000 Children Younger Than 15 Years **eFigure 7.** Association of Nonpharmaceutical Intervention Implementation and *Escherichia coli* Invasive Disease Incidence per 100 000 Children Younger Than 15 Years **eFigure 8.** Association of Nonpharmaceutical Intervention Implementation and Pneumococcal Carriage Rate of Serotype 24F in Healthy Children
eReferences
