# Supplementary material for: Association of Nonpharmaceutical Interventions During the COVID-19 Pandemic With Invasive Pneumococcal Disease, Pneumococcal Carriage, and Respiratory Viral Infections Among Children in France
Source: JAMA Netw Open. 2022 Jun 28;5(6):e2218959. doi: 10.1001/jamanetworkopen.2022.18959 (PMC9240903; doi:10.1001/jamanetworkopen.2022.18959)
Supplement: Supplement. — eTable 1. Main Nonpharmaceutical Interventions Implemented in France Against the Spread of SARS-CoV-2 During the Study Period eTable 2. Serotype Distribution Among Streptococcus pneumoniae Strains Isolated from IPD Cases and Carriage in Children eTable 3. Sensitivity Analyses of the Estimated Fraction of IPD Change After Nonpharmaceutical Interventions Associated With Changes in RSV, Influenza, and Pneumococcal Carriage eFigure 1. Association of Nonpharmaceutical Intervention Implementation and Incidence of Pediatric Invasive Pneumococcal Disease in Children Younger Than 15 Years eFigure 2. Association of Nonpharmaceutical Intervention Implementation and Incidence of Pediatric Emergency Visits for Influenza-like Illness in Children Younger Than 15 Years eFigure 3. Association of Nonpharmaceutical Intervention Implementation and Percentage of RSV-Positive Tests eFigure 4. Autocorrelation Function of Residuals, Partial Autocorrelation Function of Residuals, Standardized vs Fitted Values, and Normal Q-Q Plot of the Model Used to Estimate Change in IPD Incidence After Nonpharmaceutical Intervention Implementation eFigure 5. Autocorrelation Function of Residuals, Partial Autocorrelation Function of Residuals, Standardized vs Fitted Values, and Normal Q-Q Plot of the Model Used to Estimate Change in Pneumococcal Carriage After Nonpharmaceutical Intervention Implementation eFigure 6. Association of Nonpharmaceutical Intervention Implementation With IPD Incidence Associated With Serotype 24F per 100 000 Children Younger Than 15 Years eFigure 7. Association of Nonpharmaceutical Intervention Implementation and Escherichia coli Invasive Disease Incidence per 100 000 Children Younger Than 15 Years eFigure 8. Association of Nonpharmaceutical Intervention Implementation and Pneumococcal Carriage Rate of Serotype 24F in Healthy Children eReferences [file jamanetwopen-e2218959-s001.pdf]

Supplementary Online Content

Rybak A, Levy C, Angoulvant F, et al. Association of nonpharmaceutical interventions during the COVID-19 pandemic with invasive pneumococcal disease, pneumococcal carriage, and respiratory viral infections among children in France. *JAMA Netw Open*. 2022;5(6):e2218959. doi:10.1001/jamanetworkopen.2022.18959

eTable 1. Main Nonpharmaceutical Interventions Implemented in France Against the Spread of SARS-CoV-2 During the Study Period

eTable 2. Serotype Distribution Among *Streptococcus pneumoniae* Strains Isolated from IPD Cases and Carriage in Children

eTable 3. Sensitivity Analyses of the Estimated Fraction of IPD Change After Nonpharmaceutical Interventions Associated With Changes in RSV, Influenza, and Pneumococcal Carriage

eFigure 1. Association of Nonpharmaceutical Intervention Implementation and Incidence of Pediatric Invasive Pneumococcal Disease in Children Younger Than 15 Years

eFigure 2. Association of Nonpharmaceutical Intervention Implementation and Incidence of Pediatric Emergency Visits for Influenza-like Illness in Children Younger Than 15 Years

eFigure 3. Association of Nonpharmaceutical Intervention Implementation and Percentage of RSV-Positive Tests

eFigure 4. Autocorrelation Function of Residuals, Partial Autocorrelation Function of Residuals, Standardized vs Fitted Values, and Normal Q-Q Plot of the Model Used to Estimate Change in IPD Incidence After Nonpharmaceutical Intervention Implementation

eFigure 5. Autocorrelation Function of Residuals, Partial Autocorrelation Function of Residuals, Standardized vs Fitted Values, and Normal Q-Q Plot of the Model Used to Estimate Change in Pneumococcal Carriage After Nonpharmaceutical Intervention Implementation

eFigure 6. Association of Nonpharmaceutical Intervention Implementation With IPD Incidence Associated With Serotype 24F per 100 000 Children Younger Than 15 Years

eFigure 7. Association of Nonpharmaceutical Intervention Implementation and *Escherichia coli* Invasive Disease Incidence per 100 000 Children Younger Than 15 Years

eFigure 8. Association of Nonpharmaceutical Intervention Implementation and Pneumococcal Carriage Rate of Serotype 24F in Healthy Children

eReferences

This supplementary material has been provided by the authors to give readers additional information about their work.

© 2022 Rybak A et al. *JAMA Network Open*.

eTable 1. Main Nonpharmaceutical Interventions Implemented in France Against the Spread of SARS-CoV-2 During the Study Period[1]

**Non-pharmaceutical interventions**	**Start and end dates**
**Stay home orders**	
General order	03/17/2020 to 05/11/2020<br>10/29/2020 to 12/14/2020
Partial order	05/12/2020 to 06/02/2020<br>10/17/2020 to 10/28/2020<br>12/15/2020 to 06/20/2021
**Closure of educational institutions**	
Total closure of higher education	10/27/2020 to 01/24/2021
Partial closure of higher education	03/16/2020 to 06/22/2020<br>01/25/2021 to 09/03/2021
Total closure of secondary schools	03/16/2020 to 05/10/2020
Partial closure of secondary schools.	05/11/2020 to 06/22/2020
Partial closure primary schools	06/16/2020 to 06/22/2020
Total closure of daycare or nursery	03/16/2020 to 06/01/2020
Partial closure of daycare or nursery	06/02/2020 to 06/22/2020
**Mandatory wear of protective masks**	
In all spaces	10/28/2020 to 06/16/2021
In closed spaces	05/18/2020 (ongoing)

© 2022 Rybak A et al. *JAMA Network Open*.

eTable 2. Serotype Distribution Among *Streptococcus pneumoniae* Strains Isolated from IPD Cases (n = 4412) and Carriage in Children (n = 2013)

	**PCV7 period (January 2007-May 2010)**	**Early PCV13 period (June 2011-May 2014)**	**Late PCV13 period (June 2014-May 2017)**	**NPI period (April 2020-March 2021)**
Main serotypes isolated from IPD	19A (22%)<br>1 (19%)<br>7F (17%)<br>3 (4%)<br>24F (4%)	1 (12%)<br>12F (10%)<br>24F (9%)<br>19A (8%)<br>15B/C (7%)	24F (16%)<br>15B/C (8%)<br>10A (7%)<br>12F (7%)<br>8 (6%)	24F (17%)<br>10A (14%)<br>23B (11%)<br>15B/C (9%)<br>11A (6%)
	**PCV7 period (November 2006-May 2010)**	**Early PCV13 (June 2011-May 2014)**	**Late PCV13 (June 2014-May 2017)**	**NPI period (April 2020-April 2021)**
Main serotypes isolated from carriage	19A (12%)<br>15B/C (9%)<br>6A (8%)<br>6C (7%)<br>11A (7%)	15B/C (13%)<br>23B (10%)<br>15A (8%)<br>11A (8%)<br>23A (7%)	15B/C (12%)<br>23B (10%)<br>11A (9%)<br>23A (7%)<br>35B (6%)	15B/C (15%)<br>23B (11%)<br>15A (10%)<br>23A (9%)<br>11A (7%)

Abbreviations: IPD, invasive pneumococcal disease; PCV7, 7-valent pneumococcal conjugate vaccine; PCV13, 13-valent pneumococcal conjugate vaccine

© 2022 Rybak A et al. *JAMA Network Open*.

eTable 3. Sensitivity Analyses of the Estimated Fraction of IPD Change After Nonpharmaceutical Interventions Associated With Changes in RSV, Influenza, and Pneumococcal Carriage

	Estimated fraction of IPD attributable to					
	**Influenza**		**RSV**		**Pneumococcal carriage**	
	**% (95% CI)**	**P value**	**% (95% CI)**	**P value**	**% (95% CI)**	**P value**
Main model	53 (28 to 78)	<.001	40 (15 to 65)	.002	4 (-7 to 15)	.49
Quasi-Poisson regression model using 12-month harmonic terms to predict RSV, influenza and pneumococcal carriage	52 (27 to 76)	<.001	40 (15 to 66)	.002	4 (-7 to 14)	.48
Quasi-Poisson regression model using PED visits for bronchiolitis instead of rate of RSV-positive tests	41 (15 to 68)	<.001	53 (30 to 76)	<.001	5 (-5 to 14)	.33

Abbreviations: 95% CI; 95% confidence interval, IPD, invasive pneumococcal disease; NPI, non-pharmaceutical intervention; RSV, respiratory syncytial virus; PED, pediatric emergency department

© 2022 Rybak A et al. *JAMA Network Open*.

eFigure 1. Association of Nonpharmaceutical Intervention Implementation and Incidence of Pediatric Invasive Pneumococcal Disease in Children Younger Than 15 Years

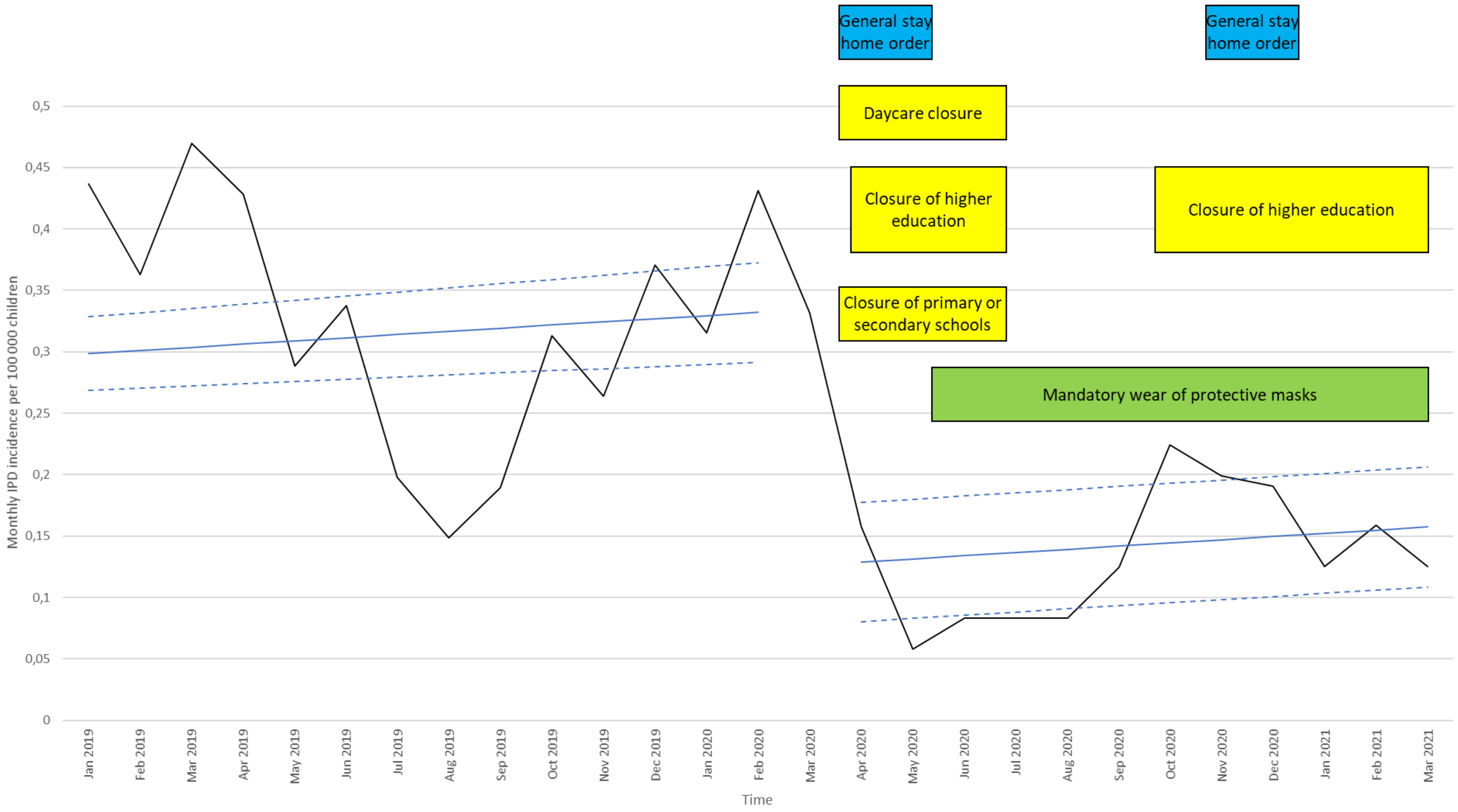

For clarity, we grouped partial and total closure of educational institutions and showed only general stay home orders. Closure of higher education and mandatory wear of protective masks continued after March 2021.

© 2022 Rybak A et al. *JAMA Network Open*.

eFigure 2. Association of Nonpharmaceutical Intervention Implementation and Incidence of Pediatric Emergency Visits for Influenza-like Illness in Children Younger Than 15 Years

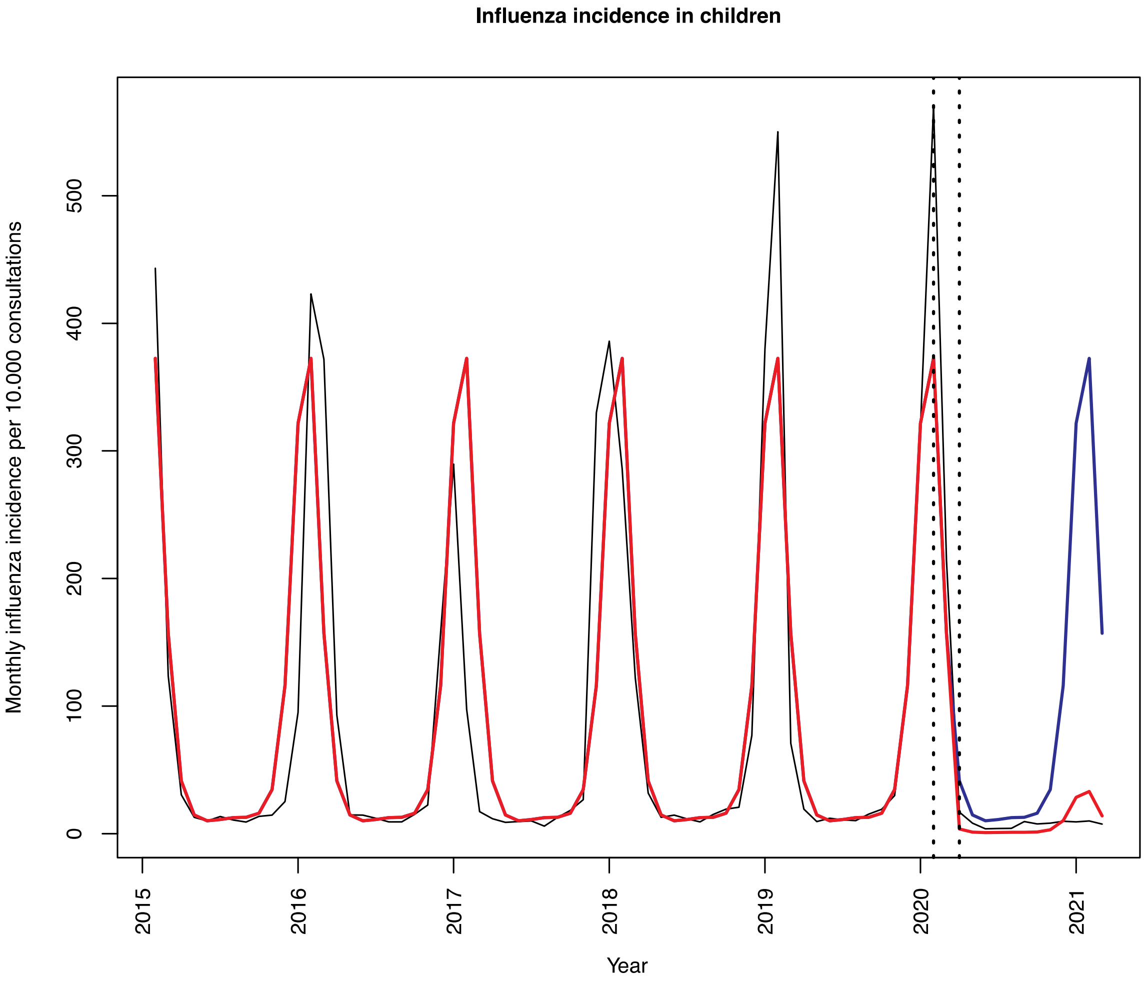

The black line shows the observed data. The red line shows the model estimates based on observed data (quasi-Poisson regression model). The blue line shows the expected values without NPIs in the post-intervention. The transition period (March 2020) when NPIs were implemented is indicated by the vertical lines.
NPI, non-pharmaceutical intervention

© 2022 Rybak A et al. *JAMA Network Open*.

eFigure 3. Association of Nonpharmaceutical Intervention Implementation and Percentage of RSV-Positive Tests

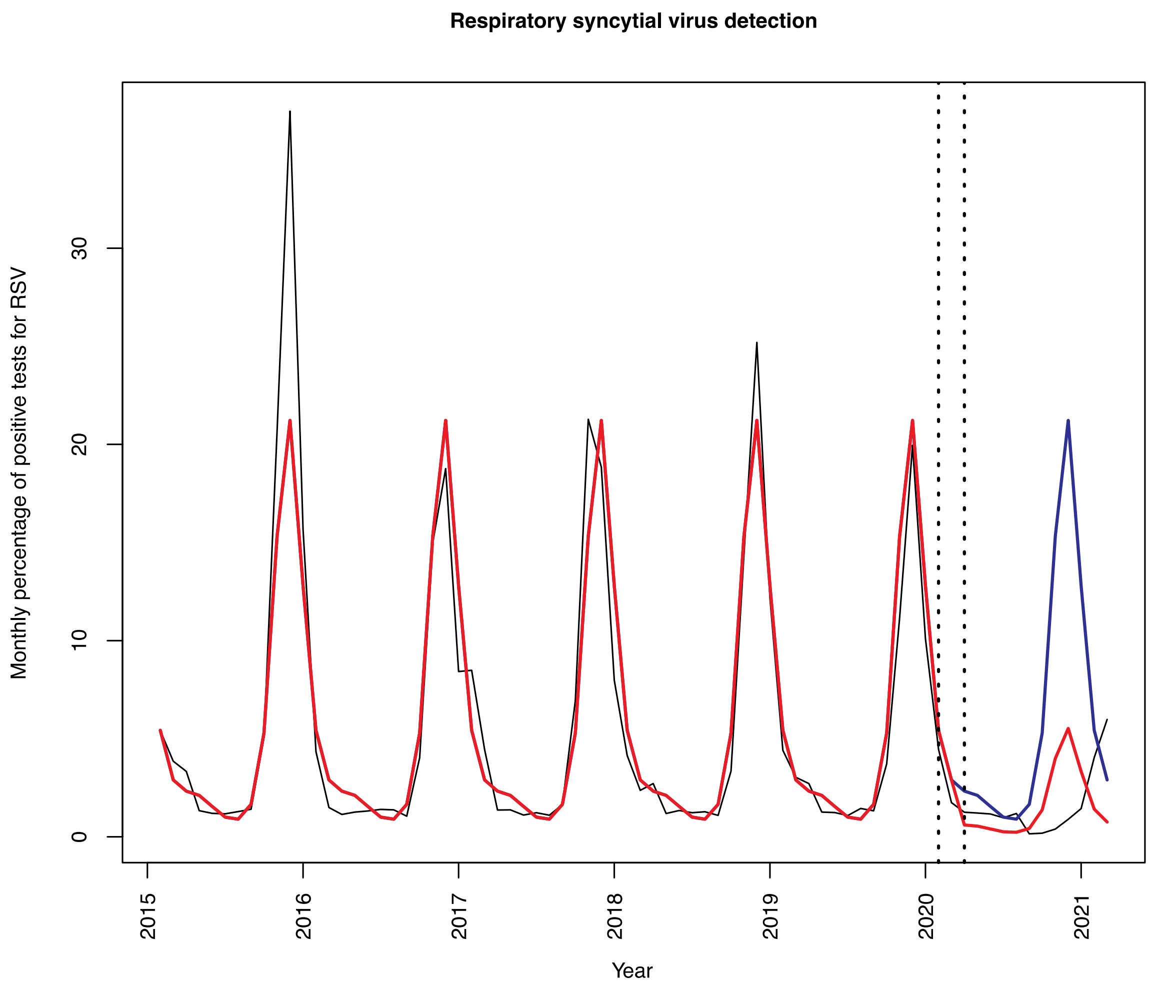

The black line shows the observed data. The red line shows the model estimates based on observed data (quasi-Poisson regression model). The blue line shows the expected values without NPIs in the post-intervention. The transition period (March 2020) when NPI were implemented is indicated by the vertical lines.
NPI, non-pharmaceutical intervention

© 2022 Rybak A et al. *JAMA Network Open*.

eFigure 4. Autocorrelation Function of Residuals, Partial Autocorrelation Function of Residuals, Standardized vs Fitted Values, and Normal Q-Q Plot of the Model Used to Estimate Change in IPD Incidence After Nonpharmaceutical Intervention Implementation

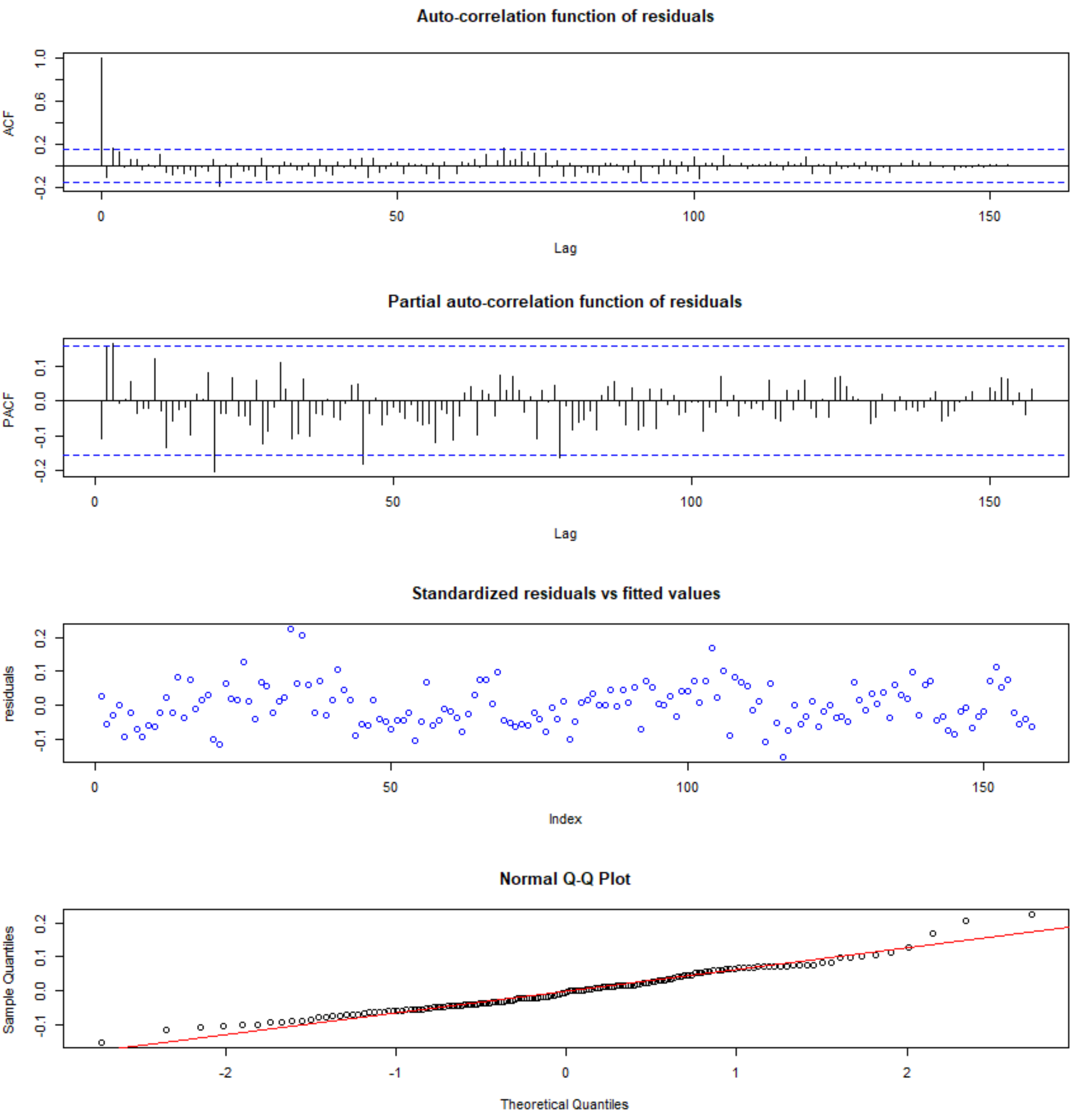

IPD, invasive pneumococcal disease; NPI, non-pharmaceutical intervention

To assess the quality of the Quasi-Poisson model, we used correlograms (autocorrelation and partial autocorrelation functions which measure the linear relationship between lagged values of a time series) and residuals analysis.
The inspection of the correlograms relies on identifying remaining autocorrelation or seasonal pattern of the residuals. The significance of any remaining autocorrelation or seasonality is defined by a correlation higher than +1.96 standard error or lower than – 1.96 standard error for each lag of the time series[2].
We checked whether the residuals of the models were normally distributed and had a constant variance over time. The correlograms were satisfactory (no remaining autocorrelation nor seasonal pattern of the residuals).

© 2022 Rybak A et al. *JAMA Network Open*.

eFigure 5. Autocorrelation Function of Residuals, Partial Autocorrelation Function of Residuals, Standardized vs Fitted Values, and Normal Q-Q Plot of the Model Used to Estimate Change in Pneumococcal Carriage After Nonpharmaceutical Intervention Implementation

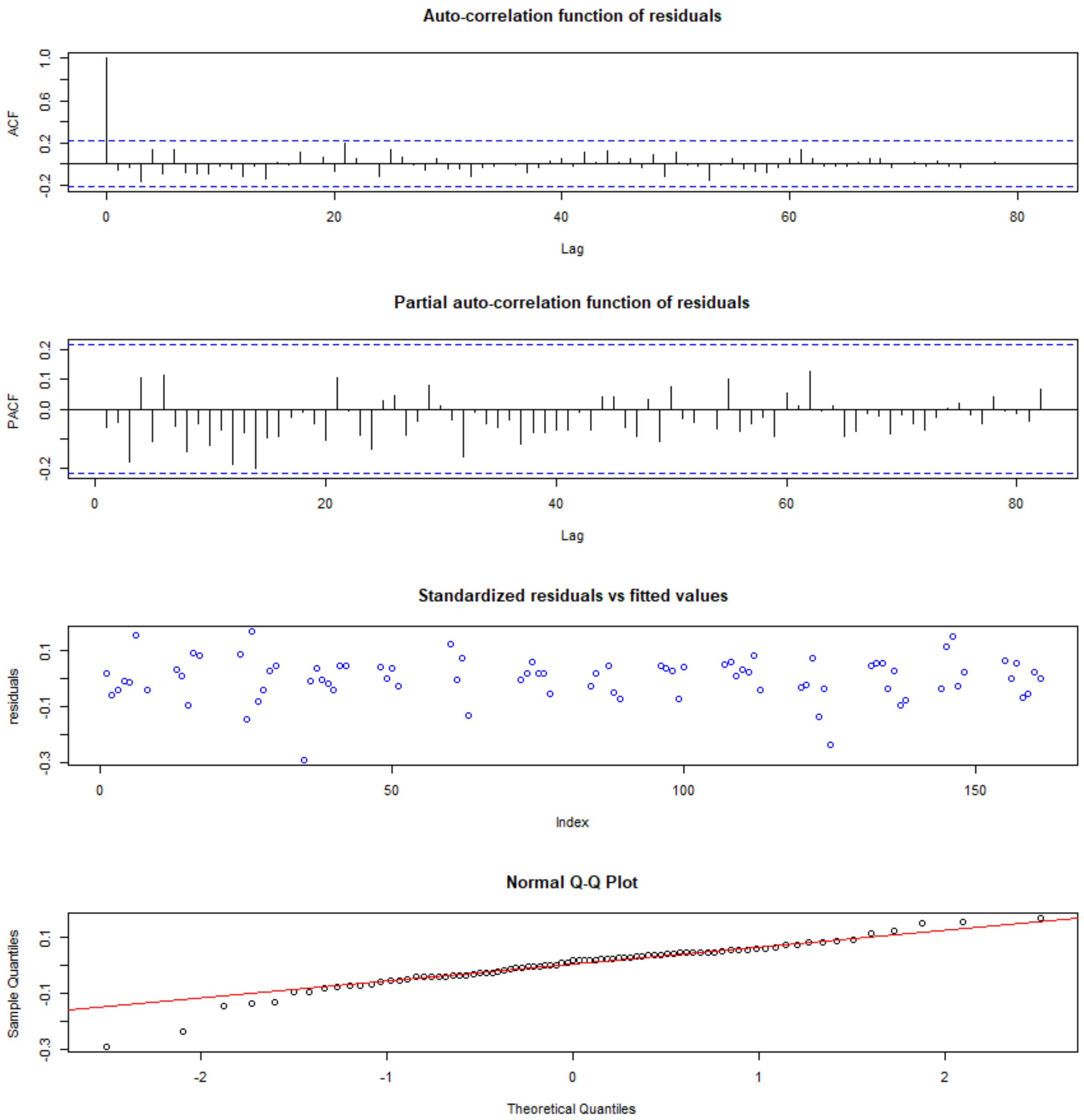

NPI, non-pharmaceutical intervention

To assess the quality of the Quasi-Poisson model, we used correlograms (autocorrelation and partial autocorrelation functions which measure the linear relationship between lagged values of a time series) and residuals analysis.
The inspection of the correlograms relies on identifying remaining autocorrelation or seasonal pattern of the residuals. The significance of any remaining autocorrelation or seasonality is defined by a correlation higher than +1.96 standard error or lower than – 1.96 standard error for each lag of the time series[2].
We checked whether the residuals of the models were normally distributed and had a constant variance over time. The correlograms were satisfactory (no remaining autocorrelation nor seasonal pattern of the residuals).

© 2022 Rybak A et al. *JAMA Network Open*.

eFigure 6. Association of Nonpharmaceutical Intervention Implementation With IPD Incidence Associated With Serotype 24F per 100 000 Children Younger Than 15 Years (n = 619)

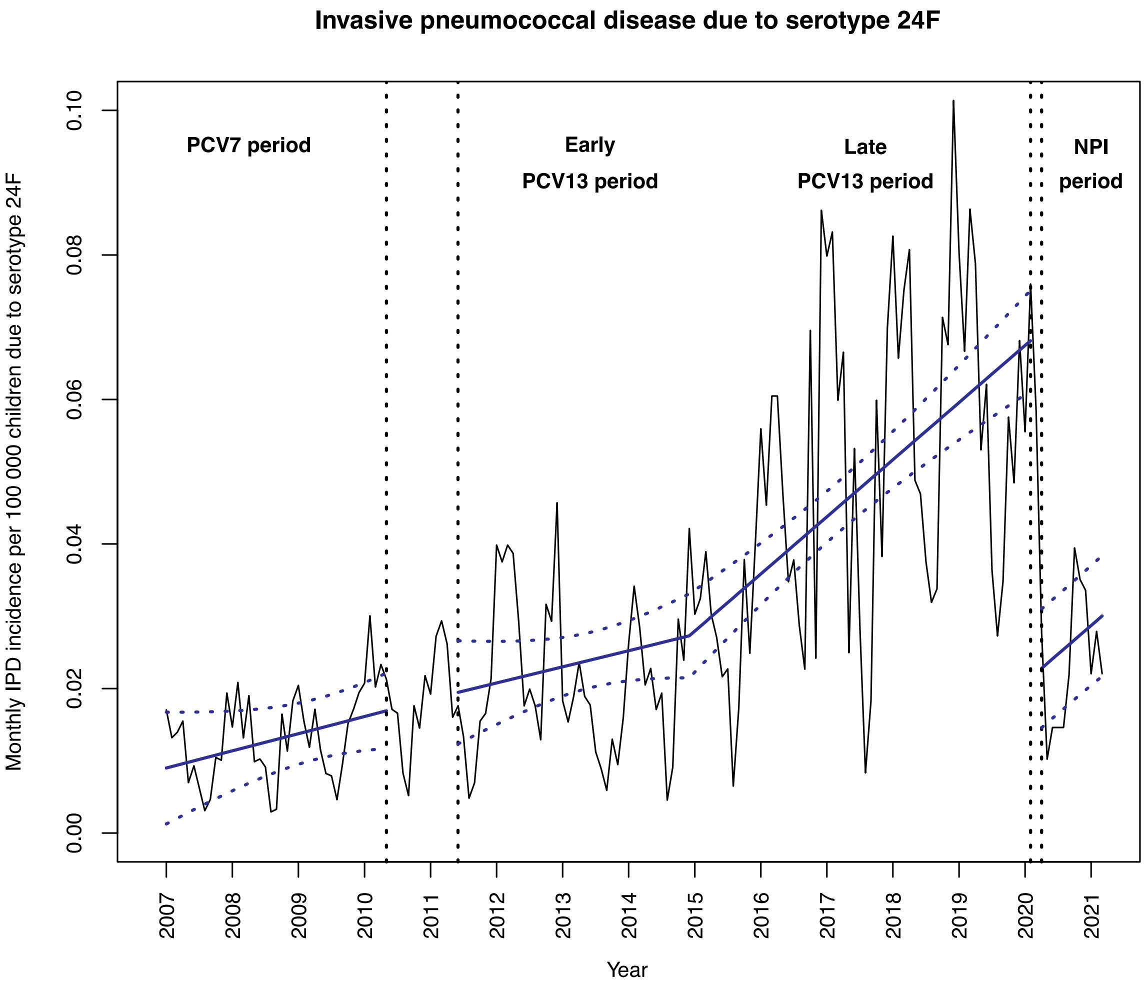

The blue slope lines were estimated by the segmented regression model. The blue dashed lines show the 95% confidence intervals (CIs) estimated by the segmented regression model. The PCV7 period was January 2007 through May 2010; the early PCV13 period, June 2011 through May 2014; the late PCV13 period, June 2014 through February 2020; and the NPI period, April 2020 to March 2021. The vertical lines show the transition period of PCV13 implementation (April 2010 through May 2011) and NPI implementation (March 2020). IPD, invasive pneumococcal disease; NPI, non-pharmaceutical intervention.

© 2022 Rybak A et al. *JAMA Network Open*.

eFigure 7. Association of Nonpharmaceutical Intervention Implementation and *Escherichia coli* Invasive Disease Incidence per 100 000 Children Younger Than 15 Years (n = 13 234)

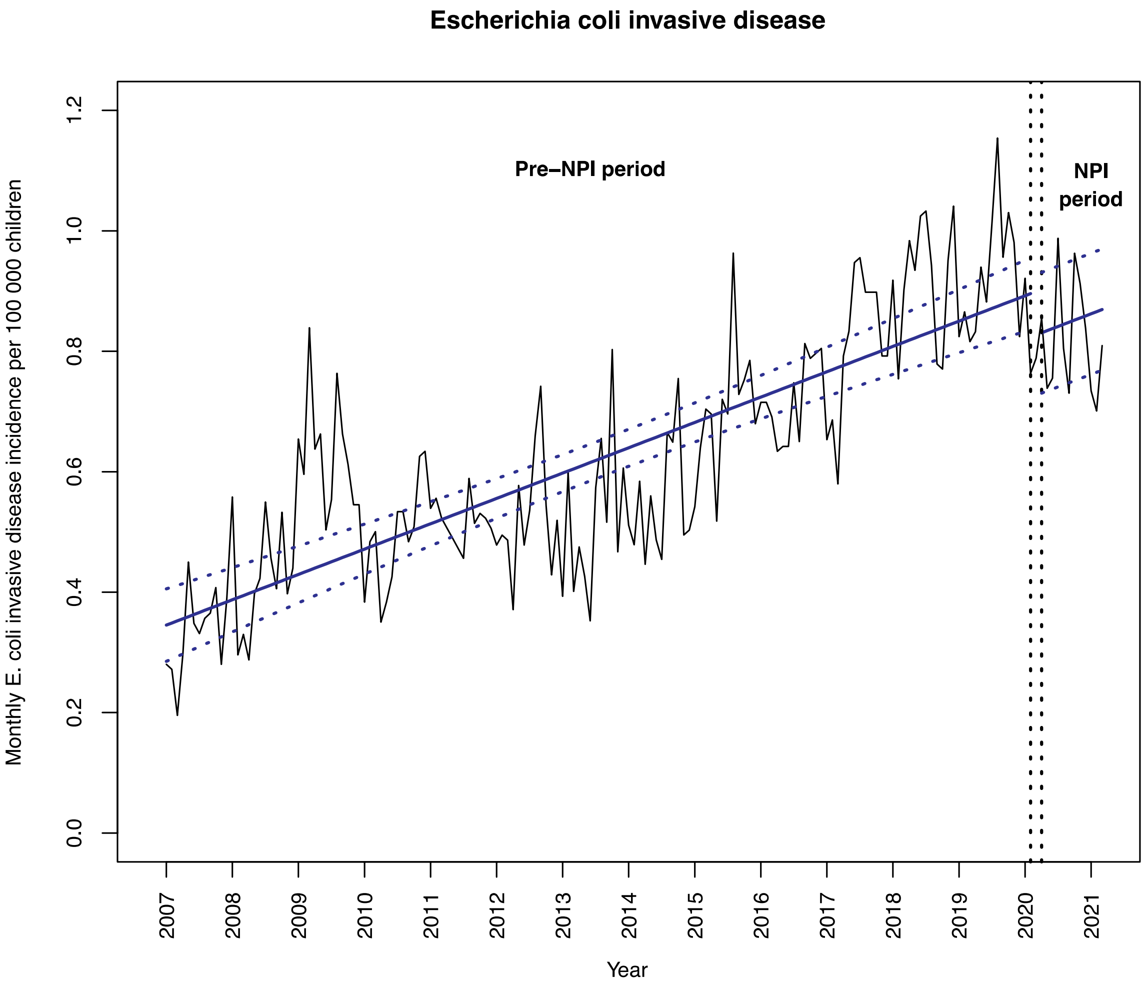

The blue slope lines were estimated by the segmented regression model. The blue dashed lines show the 95% confidence intervals (CIs) estimated by the segmented regression model. The pre-NPI period was January 2007 through February 2020 and the NPI period, April 2020 to March 2021. The vertical lines show the transition period of NPI implementation (March 2020).

© 2022 Rybak A et al. *JAMA Network Open*.

eFigure 8. Association of Nonpharmaceutical Intervention Implementation and Pneumococcal Carriage Rate of Serotype 24F in Healthy Children (n = 6831)

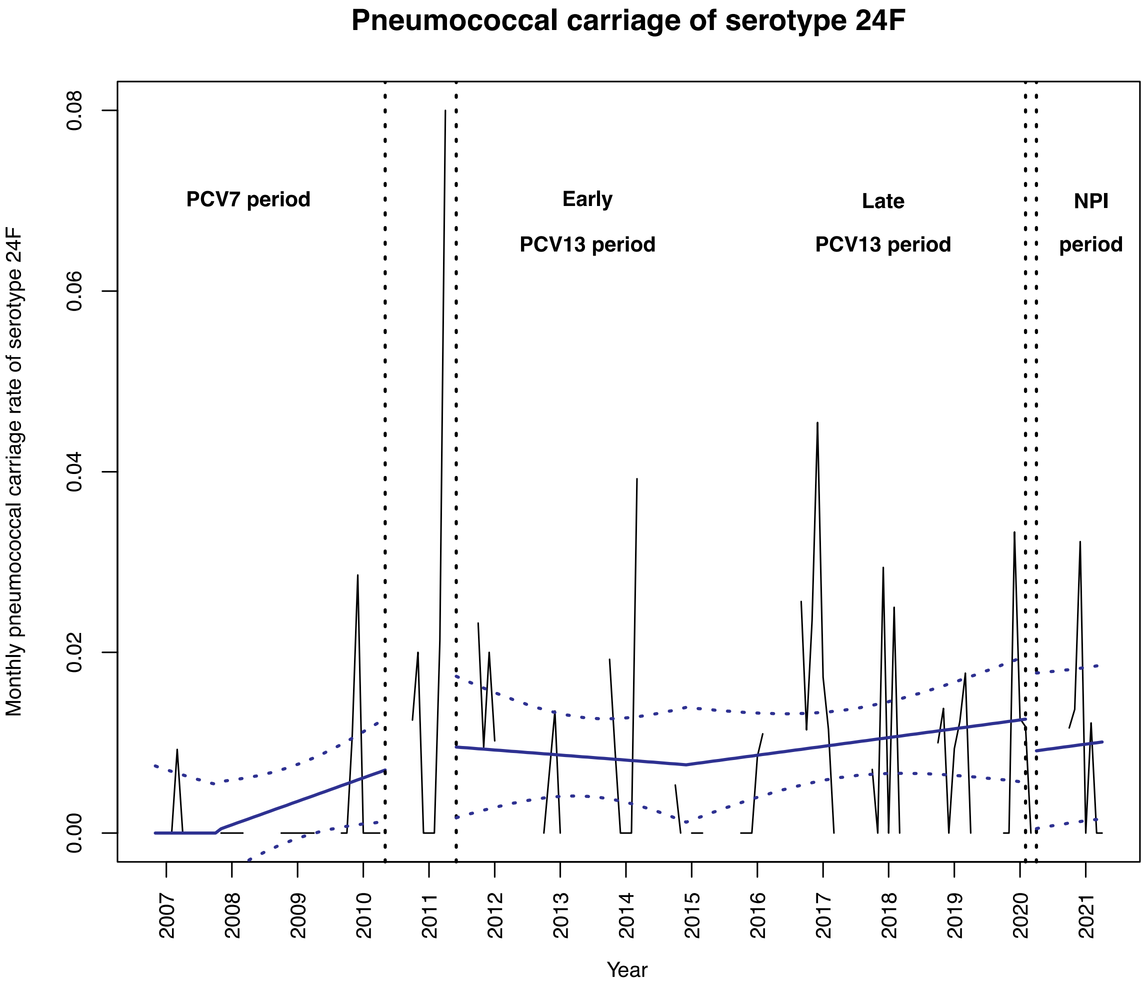

The blue slope lines were estimated by the segmented regression model. The blue dashed lines show the 95% CIs estimated by the segmented regression model. The PCV7 period was November 2006 through May 2010; the early PCV13 period, June 2011 through May 2014; the late PCV13 period, June 2014 through February 2020; and the NPI period, April 2020 to April 2021. The vertical lines show the transition period of PCV13 implementation (April 2010 through May 2011) and NPI implementation (March 2020).
NPI, non-pharmaceutical intervention.

© 2022 Rybak A et al. *JAMA Network Open*.

eReferences

1. European Centre for Disease Prevention and Control. Data on country response measures to COVID-19. https://www.ecdc.europa.eu/en/publications-data/download-data-response-measures-covid-19. Accessed 2 November, 2021.
2. Bernal JL, Cummins S, Gasparrini A. Interrupted time series regression for the evaluation of public health interventions: a tutorial. *Int J Epidemiol.* 2017;46(1):348-355.

© 2022 Rybak A et al. *JAMA Network Open*.
